# Concomitant Bladder Neck Incision in Patients with Posterior Urethral Valve and Bladder Neck Hypertrophy: Short-term Outcomes of a Randomized Controlled Trial

**DOI:** 10.1590/S1677-5538.IBJU.2025.0539

**Published:** 2026-01-28

**Authors:** Ahmed ELghareeb, Mohamed Dawaba, Mona Eldeeb, Abdelwahab Hashem, El-husseiny I. Ibrahim, Ahmed Abdelhalim

**Affiliations:** 1 Mansoura University Mansoura Urology and Nephrology Center Department of Urology Mansoura Egypt Department of Urology, Mansoura Urology and Nephrology Center, Mansoura University, Mansoura, Egypt; 2 Mansoura University Mansoura Urology and Nephrology Center Department of Radiology Mansoura Egypt Department of Radiology, Mansoura Urology and Nephrology Center, Mansoura University, Mansoura, Egypt; 3 Delta University for Science and Technology Faculty of Medicine Dakahlia Egypt Faculty of Medicine, Delta University for Science and Technology, Dakahlia, Egypt; 4 West Virginia University Department of Urology Morgantown WV USA Department of Urology, West Virginia University, Morgantown, WV, USA.

**Keywords:** Urinary Bladder Neck Obstruction, Reoperation, Ablation Techniques

## Abstract

**Introduction:**

Concomitant bladder neck incision (BNI) with posterior urethral valve ablation (VA) was proposed to mitigate the long-term sequela of posterior urethral valve (PUV) and reduce the reoperation rates. This study aimed to investigate the short-term outcomes of concomitant BNI and VA, particularly short-term reoperation rates.

**Patients and Methods:**

Patients with PUV and bladder neck hypertrophy on preoperative imaging were randomized to undergo VA only or VA with concomitant BNI. Surgical reoperation within one year was the primary endpoint. Renal function, UTI, hydronephrosis and VUR improvement at one year were secondary endpoints.

**Results:**

Sixty-three patients were included in the final analysis, 33 in VA group (group A) and 30 in concomitant BNI and VA group (group B). After one year of follow-up, the reoperation rate was similar [5(15.2%) in group A and 3(10%) in group B, p=0.18]. The median (IQR) nadir serum creatinine was lower in group B [0.2 (0.1-0.3) vs. 0.2 (0.2-0.4) mg/dL in group A, p=0.049]. The last follow-up serum creatinine median (IQR) eGFR [107 (89.5-163) in group A vs. 139(102-165) mL/min/1.73 m2 in groups B, p=0.37], and febrile UTI rates were not different between the two groups. Hydronephrosis improved/ resolved in 27 (40.9%) renal units in group A vs. 33 (55%) renal units in group B (p=0.286). Vesicoureteral reflux improved/ resolved in 23(34.8%) and 12 (20%) renal units in group A and B, respectively (p=0.074).

**Conclusion:**

Concomitant BNI with VA does not confer a lower short-term reoperation rate or better upper urinary tract outcomes compared to VA only.

## INTRODUCTION

Despite our improved understanding and the proactive management of bladder dysfunction in patients with posterior urethral valves (PUV), the long-term morbidity remains substantially high with 20-30% of patients progressing to end-stage renal disease in adolescence or early adulthood (1, 2). Bladder neck obstruction was hypothesized as one of the factors contributing to the bladder dysfunction seen in most patients after valve ablation (VA). Alpha-blocker treatment, clean intermittent catheterization (CIC), overnight bladder drainage, and bladder neck incision (BNI) were proposed as treatment options (2–4). BNI is viewed as the most definitive treatment of bladder neck obstruction with controversial benefits in PUV population. Kajbafzadeh et al. reported decreased long-term need for anticholinergics and CIC when BNI was concomitantly performed with VA. Further, BNI was associated with reduced short-term reoperation rates in their cohort. No short-term reinterventions were required in their cohort when BNI was concurrently done with VA compared to 24% reintervention rate in patients treated with VA only (5). The short-term benefits of BNI in PUV population is as debatable as its long-term gains. In a retrospective study by Abdelhalim et al., the short-term reoperation rate was not different among patients treated with VA or concomitant BNI and VA (6). To solve this controversy, this randomized controlled study was conducted to assess the effect of concomitant BNI on the short-term reoperation rates in patients with PUV. We hypothesize that concomitant BNI and VA are associated with less short-term reoperation rates than VA only.

## PATIENTS AND METHODS

### Patients

The study was approved by the Institutional Review Board (MS.21.09.1655) and was registered on ClinicalTrials.gov (NCT05087537). Patients younger than 12 years diagnosed with PUV at a single tertiary center between January 2020 and January 2022, were screened for eligibility. Patients were considered eligible if they had evidence of bladder neck hypertrophy, defined as bladder neck shouldering on preoperative VCUG. Bladder neck hypertrophy was confirmed by visualizing elevated posterior lip of the bladder neck on cystoscopic examination (7). Patients were excluded if they had prior surgical treatment for PUV. Parents/ legal guardians of eligible patients were approached by the study team, and the study methodology was thoroughly explained. The pros and cons of each treatment approach were discussed. Parents who agreed to enroll their children in the study provided informed consents. Patients were randomly assigned to one of the treatment groups using the closed envelop method in a 1:1 ratio. Patients in group A were managed with endoscopic VA only, whereas group B patients were treated with concomitant VA and BNI. Surgeries were conducted by one of three fellowship-trained pediatric urologists.

### Baseline evaluation

Baseline evaluation included history, physical exam, and serum chemistry with calculation of the estimated glomerular filtration rate (eGFR) using the modified Schwartz formula (8), renal bladder ultrasound, and VCUG.

### Surgical technique

**Endoscopic valve ablation:**

Transurethral VA was done under direct vision using the pediatric cold knife urethrotome. The valve leaflets were incised at 5, 7, and 12 o'clock positions.

**Bladder neck incision:**

In children assigned to concomitant BNI, an additional single incision through the bladder neck was made at 6 o'clock until the bladder lumen was visible with the tip of the scope at the verumontanum (6). In both groups, a Foley catheter was left for 24-48 hours.

### Follow-up

Patients were followed up every 3 months for at least one year. Follow-up entailed history with emphasis on febrile UTI. Laboratory evaluation included serum creatinine with calculation of eGFR. Follow-up imaging included renal bladder ultrasound every 3 months. Hydronephrosis severity was graded according to the Society of Fetal Urology (SFU) Hydronephrosis grading system (9) and measuring the antero-posterior diameter of the renal pelvis. VCUG was repeated at 3 and 12 months postoperatively. Patients with high-grade vesicoureteral reflux (VUR) or high-grade hydronephrosis were maintained on continuous antibiotic prophylaxis. Oxybutynin treatment was selectively considered for patients with non-improving hydronephrosis in the absence of anatomic obstruction (10). Alpha-blocker treatment was not given to any of the study participants to avoid its confounding effects.

### Study outcomes

**Primary study outcome:**

Surgical reoperation rates within one year of the primary surgery, including check cystoscopy with or without the need for ablation of valve remnants, BNI, or urinary diversion. The clinical indications for reoperation were recurrent febrile UTI, weak urine stream or repeated urinary retention. The laboratory indications for reoperation were renal functional deterioration in the absence of radiological improvement manifested by non-improved hydronephrosis or VUR, or persistent dilation of the posterior urethra on follow-up VCUG.

**Secondary study outcomes:**

- Renal function outcomes: nadir serum creatinine (lowest serum creatinine within one year of surgery), serum creatinine, eGFR at 12 months of follow-up, progression to chronic kidney disease defined as eGFR ≤ 60 mL/min/1.73 m2.
- Febrile UTIs defined as fever ≥ 38° C in with a positive urinalysis and a positive urine culture of an appropriately collected urine specimen.
- Hydronephrosis improvement, defined as complete hydronephrosis resolution or improvement by one or more grades according to the SFU grading system.
- VUR improvement, defined as complete VUR resolution or downgrading by one or more grades on VCUG according to the International Reflux Study Grading System.

### Sample size

The study sample size was calculated based on a previous study (11) in which reoperation was needed in 24% of PUV patients treated with VA only compared to 0% in those treated with concomitant VA and BNI. The G-power statistical software (Universität Düsseldorf) was used with an effect size of 24%, alpha error 0.05, study power 0.80, and an expected dropout rate of 10%. The total sample size was 56 patients, 28 patients in each study group.

### Statistical Analysis

Statistical analysis was done by the Statistical Package for Social Sciences "IBM SPSS Statistics (Version 27)". Numbers and percentages were used to describe categorical data. Quantitative data was presented as medians and interquartile ranges. Mann–Whitney test was used to compare continuous variables and Chi‐squared or Fisher's exact tests for the categorical variables. P value ≤ 0.05 was used to indicate statistical significance. Outcomes were analyzed based on the intention to treat.

## RESULTS

### Study enrollment

Patients were recruited for participation in the study from January 2020 to January 2022. Out of 93 screened PUV patients, 16 patients did not meet the inclusion criteria (10 had prior surgical treatment of PUV, and 6 did not have high bladder neck on VCUG) and were excluded. Nine other patients refused to participate in the study.

Of the 68 patients enrolled in the study, 34 patients were assigned to group A (primary VA only) and 34 to group B (concomitant VA and BNI). After surgical intervention, one patient in group (A) and three patients in group (B) lost follow-up and were excluded from the analysis. Another group B patient opted out of the study during follow-up.

**Figure 1:**
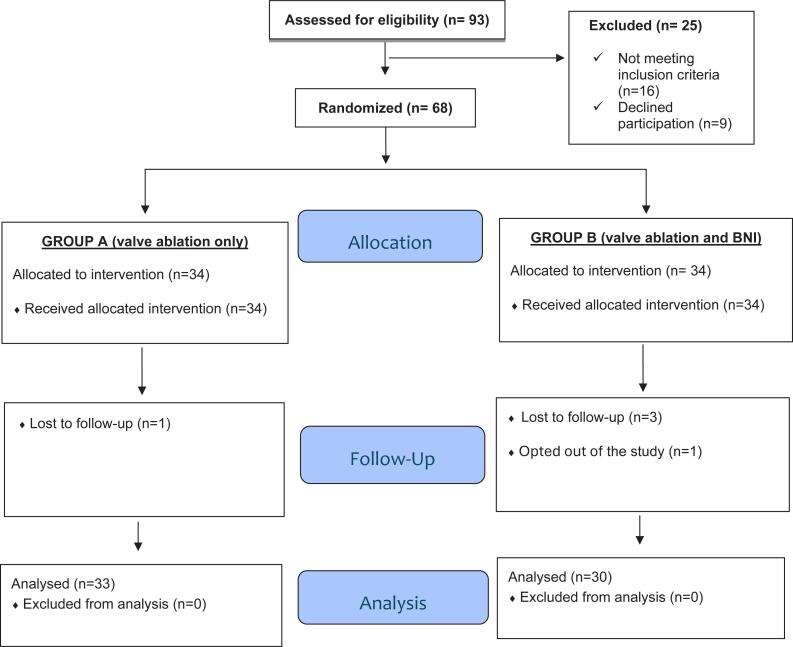
The study CONSORT flowchart.

A total of 63 patients were included in the final analysis: 33 patients in VA (group A) and 30 patients in combined VA and BNI group (group B). The enrollment process is summarized in Figure-1.

### Baseline demographics

Patients in group B were relatively younger. The median age at surgery was 4 and 1.75 months in groups A and B, respectively (p = 0.045). Otherwise, baseline demographics were similar between the study groups (Table-1). Overall, 37/63 (58.7%) of patients were suspected on prenatal imaging, 11 (17.5%) presented with febrile UTI, and 8 (12.7%) with urinary retention or urine stream abnormalities. Bilateral hydronephrosis was observed in 72.7% and 80% of group A and B, respectively (p =0.379). Oxybutynin treatment was administered in 3 (10%) patients in group A and 4 (12.1%) patients in group B for non-improving hydronephrosis after excluding bladder outlet obstruction at least 3 months following primary intervention. Baseline demographics are summarized in Table-1.

**Table 1:** Baseline demographics.

Baseline demographics		Group A: Valve ablation only (n =33)	Group B: Combined valve ablation and BNI (n = 30)	P value
Median age at surgery (IQR), months		4 (1.5-21.5)	1.75 (1-8.6)	0.045
**Presentation (%)**				
	Antenatal hydronephrosis	18 (54.5)	19 (63.3)	
	Febrile UTI	7 (21.2)	4 (13.3)	
Urine retention/ abnormal urine stream		6 (18.2)	2 (6.7)	0.452
Postnatal hydronephrosis		2 (6.1)	5 (16.7)	
**Valve type (%)**				
	Type I	31 (93.9)	29 (96.7)	
	Type III	2 (6.1)	1 (3.3)	0.612
Median baseline serum creatinine (IQR), mg/dL (mg/dL)		0.4 (0.3-0.8)	0.5 (0.3-0.73)	0.825
Median baseline eGFR (IQR), (mL/min/1.73 m^2^)		66 (35.5-119)	59 (30.5-85.8)	0.401
Renal units with baseline hydronephrosis (%)		55/66 (83.3)	54/60 (90)	0.274
Baseline hydronephrosis laterality (%)				0.379
**No**		2 (6.1)	0	
	Unilateral	7 (21.2)	6 (20)	
	Bilateral	24 (72.7)	24 (80)	
**Baseline highest grade of hydronephrosis (%)**				0.073
	No	2 (6.1)	0	
	Grade I	5 (15.2)	2 (6.7)	
	Grade II	11 (33.3)	7 (23.3)	
	Grade III	14 (42.4)	14 (46.7)	
	Grade IV	1 (3)	7 (23.3)	
Renal units with baseline vesicoureteral reflux (%)		31/66 (47)	21/60 (35)	0.137
**Baseline vesicoureteral reflux laterality (%)**				0.309
	No	13 (39.4)	14 (46.7)	
	Unilateral	9 (27.3)	11 (36.7)	
	Bilateral	11 (33.3)	5 (16.7)	
**Baseline highest grade of vesicoureteral (%)**				0.440
	No	13 (39.4)	14 (46.7)	
	Grade I	0	0	
	Grade II	1 (3)	0	
	Grade III	0	0	
	Grade IV	4 (12.1)	1 (3.3)	
	Grade V	15 (45.5)	15 (50)	

p-value is in bold when differences were significant

### Study outcomes

The study outcomes are summarized in Table-2.

**Table 2:** Study outcomes at 12 months.

Study outcomes	Group A: Valve ablation only (n =33)	Group B: Combined valve ablation and BNI (n = 30)	P value
Overall reintervention (%)	5 (15.2)	3 (10)	
Check cystoscopy only	1 (3)	3(10)	
Ablation of valve remnants	0 (0)	0(0)	0.18
BNI	3 (9)	0(0)	
Ablation of valve remnants and BNI	1 (3)	0(0)	
Median nadir serum creatinine (IQR), mg/dL	0.2 (0.2 - 0.4)	0.2 (0.1 - 0.3)	0.049
Median serum creatinine at 12 months (IQR), mg/dL	0.3 (0.2 - 0.45)	0.2 (0.2 - 0.4)	0.09
Median eGFR (IQR) at 12 months, mL/min/1.73 m^2^	107 (89.5-163)	139 (102-165)	0.374
Febrile UTI during follow up, patients (%)	9 (27.3)	7 (23.3)	0.778
Renal units with improved/ resolved hydronephrosis (%)	27/66 (40.9%)	33/60 (55%)	0.286
Renal units with improved/ resolved VUR (%)	23/66 (34.8%)	12/60 (20%)	0.074

p-value is in bold when differences were significant

- **Reoperation rate within one year of follow-up:**
  Five patients (15.2%) in group A and 3 (10%) in group B required reoperation within one year of follow-up (p= 0.18). All patients who required reintervention were symptomatic. In group A, the indications for reintervention were breakthrough UTI in 3 patients, difficulty and a weak urinary stream in one patient, and UTI with a weak urine stream in one patient. One patient had diagnostic cystoscopy with no evidence of bladder outlet obstruction, 3 had BNI, and one had ablation of valve remnants and BNI as salvage treatment for symptomatic patients. The indications for intervention in group B patients were recurrent breakthrough UTI in two patients and repeated urinary retention in one patient. All 3 patients in group B who required reoperation had check cystoscopies that ruled out anatomic bladder outlet obstruction, and no further intervention was deemed necessary.
- **Renal function outcomes**
  The median (IQR) nadir serum creatinine was lower in group B [0.2 (0.1-0.3) mg/dL vs 0.2 (0.2-0.4) mg/dL in group A, p=0.049]. The median serum creatinine and eGFR at 12 months of follow-up were not significantly different between the study groups. Five (15.2%) group A and 3 (10%) group B patients had eGFR < 60 mL/min/1.73 m2 at 12 months (Fisher's exact p = 0.710).
- **Febrile UTI**
  Nine patients (27.3%) in group A and 7 (23.3%) in group B had febrile UTIs during follow up (p = 0.778).
- **Follow-up imaging**
  Twenty-seven (40.9%) renal units in group A and 33 (55%) renal units in group B had improved/resolved hydronephrosis (p= 0.286). Vesicoureteral reflux improved/ resolved in 23 (34.8%) and 12 (20%) renal units in groups A and B, respectively (p = 0.074).

## DISCUSSION

Following endoscopic ablation of PUV, vigilant monitoring and proactive management of the underlying bladder dysfunction are the pillars of modern urologic management of PUV. Left untreated, bladder dysfunction contributes to hydronephrosis and VUR persistence, increased risk of UTI and incontinence, and accelerates renal damage and progression to end-stage renal disease. In 1982, Dr. Mitchell coined the term valve bladder syndrome to describe the bladder dysfunction seen in 75-80% of patients following PUV ablation (12). Some of the theorized mechanisms for valve bladder syndrome are detrusor hypertrophy, increased extracellular matrix deposition, bladder wall ischemia, diminished bladder sensations, incomplete bladder emptying, and high urine output resulting from poor renal tubular concentration capacity. In addition to these possible causes, bladder neck obstruction caused by blader neck hypertrophy or dyskinesis can interfere with bladder emptying and contribute to elevated bladder pressures, detrusor decompensation and eventually myogenic failure (2, 7).

The diagnosis of bladder neck obstruction in children with PUV is challenging. To date, there is no consensus on how to define bladder outlet obstruction in children (13). As in this study, the radiologic and endoscopic appearance of the bladder neck was used by some investigators to diagnose bladder neck hypertrophy (7, 14). Glassberg and Combs believed that diagnosing bladder neck obstruction requires videourodynamic documentation of elevated voiding pressure, and obstructed uroflow with a silent electromyogram (3).

Alpha blockers, CIC, overnight bladder drainage, bladder neck botulinum toxin injection, and BNI were proposed as therapeutic options for bladder neck obstruction in PUV with varying results and limitations (2, 5, 6, 15, 16). For instance, some studies reported subjective improvement of the voiding pattern with decreased maximum voiding detrusor pressure, increased Qmax, and reduced postvoid residual (PVR) with alpha blocker treatment (4, 17, 18). Bajpai reported improved radiological appearance of BN hypertrophy, reduced PVR, and increased bladder capacity following prazosin treatment in PUV patients (19). Mendez-Serrano reported improved hydronephrosis and decreased risk of progression of chronic kidney disease when comparing patients treated with and without alpha blockers (20). Conversely, botulinum toxin injection into the bladder neck of patients with blader neck dysfunction following VA failed to improve urodynamic parameters, hydronephrosis or VUR resolution in a study by Mokhless et al. (15). Likewise, Sarin et al. failed to demonstrate any urodynamic benefit when BNI was combined with VA (14). Singh et al. reported improved Qmax and PVR with concomitant BNI, but similar compliance, detrusor overactivity, end-filling detrusor pressure, maximum Pdet at Qmax and VUR resolution rates in a prospective randomized study (21).

BNI is considered the most definitive treatment of bladder neck obstruction and was widely practiced in patients with PUV in the 1950s. This practice was later abandoned for fear of incontinence and risk of retrograde ejaculation (22). Concomitant VA and BNI was proposed as one-stop treatment to relieve bladder outlet obstruction, dramatically decrease voiding pressures, and decompress the dilated upper tracts. Recent data showing no ill effects of BNI on continence and antegrade ejaculation provided assurance to advocates of this approach. In a comparative study, 22 patients treated with VA and BNI had a significantly lower maximal voiding pressure (53±15cm H2O) and no detrusor overactivity, whereas 24 patients treated with VA only had Pdet maximal voiding pressure of 87±45cm H2O and 25% had detrusor overactivity. These favorable urodynamic effects were associated with less long-term need for anticholinergic treatment and CIC in patients treated with concomitant VA and BNI (5). Long-term follow-up of 301 patients treated with concurrent BNI and PUV ablation by the same group showed improved hydronephrosis from 88.3% at baseline to 24.3% and improved VUR from 62.5% to 6.6% after a mean follow-up of 5.1± 2.8 years. None of those patients had myogenic failure (23).

In addition to these potential long-term benefits of concomitant BNI, Kajbafzadeh et al. reported additional short-term advantage with lower rates of readmission and short-term reoperation. Patients treated with VA only had a reoperation rate of 24% compared to 0% in patients treated with concomitant BNI and VA (5). In our analysis, the reoperation rate within one year was similar with both approaches. 15% of patients treated with VA only and 10% of patients treated with concomitant VA and BNI failed to demonstrate clinical, laboratory, or radiological improvement and eventually required at least check cystoscopy to rule out residual bladder outlet obstruction. After one year of follow-up, hydronephrosis and VUR resolution or improvement were not significantly different between the two study arms. Except for a marginally lower median nadir serum creatinine in the group treated with VA and BNI, other measures of renal function outcome were not significantly different between both groups. In a similar context, reoperation rates and renal function measures were not significantly different between VA only and concomitant VA and BNI in a retrospective comparative study after a median follow-up was 58 (18-230) months (6). It is noteworthy that 4 patients in group A underwent BNI after suffering repeated retention and/or febrile UTI in the absence of other evidence of mechanical bladder outlet obstruction. The results were analyzed based on the intention to treat. The 3 patients who had reoperation in group B had check cystoscopy only. These factors could have skewed the results in favor of VA only resulting in a statistically similar reintervention rate.

Recent long-term follow-up of patients who had BNI during childhood for a variety of conditions showed no effect of BNI on continence, antegrade ejaculation or semen quality (24, 25). Hennus reported antegrade ejaculation in 40 men who had superficial BNI at a mean age of 4.5 years, 10.8% had reduced ejaculate volume and 5.8% had moderate incontinence (26). However, lack of evidence of adverse effects of BNI does not justify its routine use in the absence of compelling evidence of its beneficial effects.

Several study limitations should be acknowledged. First, the study included a small number of patients. Second, the diagnosis of bladder neck hypertrophy was based on radiological and not urodynamic evaluation. However, urodynamic testing is not commonly practiced before VA and there is no consensus on the definition of bladder outlet obstruction in infants. Should there be evidence of bladder outlet obstruction on urodynamic testing, it would be impossible to tell if it is caused by the valve leaflet or bladder neck hypertrophy before VA. Salvage BNI was performed in four patients in the VA arm who suffered repeated urine retention and/or febrile UTI. The use of the intention-to-treat analysis could have resulted in the absence of significant outcome differences. Further, the study follow-up duration is not long enough to monitor renal function outcomes in chronic diseases like PUV, but the primary study question was whether BNI reduces the need for short term reoperation. A recent meta-analysis demonstrated potential long-term effects of BNI on PUV outcomes but similar reintervention rates (27). Finally, the study lacked urodynamic evaluation at follow-up. Despite its value, urodynamics have several limitations in the PUV population including sensate urethras, difficult catheter insertion in patients with bladder neck hypertrophy, the high prevalence of high-grade VUR, and rater variability. These factors do not only increase the technical difficulty of urodynamics, but also limit the accuracy of bladder volume, pressure and compliance measurements.

## CONCLUSION

In patients with PUV, concomitant BNI and VA does not confer additional short-term benefits compared to VA only. Patients treated with concomitant BNI and VA had similar rates of short-term reoperation, UTI, hydronephrosis and VUR resolution. With short-term follow-up, renal function outcomes were similar among patients treated with VA only or with concomitant BNI.

## Data Availability

All data generated or analysed during this study are included in this published article

## ABBREVIATIONS

BNI: = bladder neck incision
CIC: = clean intermittent catheterization
eGFR: = estimated glomerular filtration rate
PUV: = posterior urethral valve
PVR: = postvoid residual
VA: = valve ablation
VUR: = vesicoureteral reflux

## References

[1] Hennus PM, van der Heijden GJ, Bosch JL, de Jong TP, de Kort LM (2012). A systematic review on renal and bladder dysfunction after endoscopic treatment of infravesical obstruction in boys. PLoS One.

[2] Abdelhalim A, Hafez AT (2021). Antenatal and postnatal management of posterior urethral valves: where do we stand?. Afr J Urol.

[3] Glassberg KI, Combs A (2016). The valve bladder syndrome: 35+ years later. J Urol.

[4] Combs AJ, Horowitz M, Glassberg KI (2009). Secondary bladder neck obstruction in boys with a history of posterior urethral valve: revisited. J Urol.

[5] Kajbafzadeh AM, Payabvash S, Karimian G (2007). The effects of bladder neck incision on urodynamic abnormalities of children with posterior urethral valves. J Urol.

[6] Abdelhalim A, Hashem A, Abouelenein EE, Atwa AM, Soltan M, Hafez AT (2022). Can Concomitant Bladder Neck Incision and Primary Valve Ablation Reduce Early Re-admission Rate and Secondary Intervention?. Int Braz J Urol.

[7] Androulakakis PA, Karamanolakis DK, Tsahouridis G, Stefanidis AA, Palaeodimos I (2005). Myogenic bladder decompensation in boys with a history of posterior urethral valves is caused by secondary bladder neck obstruction?. BJU Int.

[8] Schwartz GJ, Munoz A, Schneider MF, Mak RH, Kaskel F, Warady BA (2009). New equations to estimate GFR in children with CKD. J Am Soc Nephrol.

[9] Fernbach SK, Maizels M, Conway JJ (1993). Ultrasound grading of hydronephrosis: introduction to the system used by the Society for Fetal Urology. Pediatr Radiol.

[10] Abdelhalim A, El-Hefnawy AS, Dawaba ME, Bazeed MA, Hafez AT (2020). Effect of early oxybutynin treatment on posterior urethral valve outcomes in infants: a randomized controlled trial. J Urol.

[11] Kajbafzadeh AM, Payabvash S, Karimian G (2007). The effects of bladder neck incision on urodynamic abnormalities of children with posterior urethral valves. J Urol.

[12] Mitchell ME (1982). Persistent ureteral dilatation following valve ablation. Dialogues Pediatr Urol.

[13] Guha Vaze P, Saha S, Sinha R, Banerjee S (2021). Urodynamics in Posterior Urethral Valve: Pursuit of prognostication or optimisation. J Pediatr Urol.

[14] Sarin YK, Sinha S (2013). Efficacy of bladder neck incision on urodynamic abnormalities in patients with posterior urethral valves. Pediatr Surg Int.

[15] Mokhless I, Zahran AR, Saad A, Yehia M, Youssif ME (2014). Effect of Botox injection at the bladder neck in boys with bladder dysfunction after valve ablation. J Pediatr Urol.

[16] Elkashef A, Abdelhalim A, Dawaba MS, Hafez AT (2025). Effect of overnight bladder drainage on posterior urethral valve sequelae: a randomized controlled trial. J Pediatr Urol.

[17] Abraham MK, Nasir AR, Sudarsanan B, Puzhankara R, Kedari PM, Unnithan GR (2009). Role of alpha adrenergic blocker in the management of posterior urethral valves. Pediatr Surg Int.

[18] Aboulela WN, Eladawy MS, Latif AA (2024). The effect of use of alpha-blockers in posterior urethral valve pediatric patients postvalve ablation in the absence of further outlet obstruction. Urol Ann.

[19] Bajpai M, Baba A, Singh AK (2021). Postablation and α-1 blocker therapy in children with congenital obstructing posterior urethral membrane. Formos J Surg.

[20] Mendez-Serrano CG, Fang A, Fine R, Lee J, Brenseke W, Franco I (2024). Effects of alpha blockers on hydronephrosis and renal function in patients with posterior urethral valves. J Urol.

[21] Singh SK, Sharma V, Singh A (2019). Urodynamic changes after valve fulguration alone and valve fulguration with bladder neck incision. J Indian Assoc Pediatr Surg.

[22] Glassberg KI (2001). The valve bladder syndrome: 20 years later. J Urol.

[23] Sobhani S, Foroushani AR, Arshadi H, Hekmati P, Kajbafzadeh AM (2024). Simultaneous primary posterior urethral valves ablation and bladder neck incision may decrease kidney and bladder failure in long-term follow-up in patients with bladder neck hypertrophy and poor bladder function at presentation: report of 301 cases. BMC Urol.

[24] Keihani S, Kajbafzadeh AM, Kameli SM, Abbasioun R (2017). Long-term impacts of concurrent posterior urethral valve ablation and bladder neck incision on urinary continence and ejaculation. Urology.

[25] Taskinen S, Heikkila J, Rintala R (2012). Effects of posterior urethral valves on long-term bladder and sexual function. Nat Rev Urol.

[26] Hennus PML, Hoenjet E, Kieft JH, de Jong T, de Kort LMO (2017). The long-term effect of superficial bladder neck incision on ejaculation and incontinence in boys with primary and secondary bladder neck obstruction. Front Pediatr.

[27] Tharwat M, Ramadan R, Hashem A, Taha DE, Hussiny M, Elkashef A (2025). Bladder neck incision in posterior urethral valve management: a meta-analysis with insights into adjunctive bladder interventions. Curr Urol Rep.

